# Differences in cerebral saturation measured during prehospital advanced life support, between patients with presumed cardiac origin and noncardiac origin of cardiac arrest

**DOI:** 10.1186/cc14515

**Published:** 2015-03-16

**Authors:** C Genbrugge, W Boer, K Anseeuw, I Meex, F Jans, J Dens, C De Deyne

**Affiliations:** 1 ZOL, Genk, Belgium; 2 ZNA Stuivenberg Belgium; 3 Hasselt University, Hasselt, Belgium

## Introduction

During out-of hospital cardiac arrest (OHCA) cerebral saturation may provide relevant information on cerebral oxygenation. In this study we examined the time course in cerebral saturation (rSO_2_) during prehospital ALS comparing patients with a presumed cardiac origin (survivor = Sc, nonsurvivor = NSc) of arrest and noncardiac origin (survivor = Snc, nonsurvivor = NSnc) of arrest.

## Methods

With IRB approval, we prospectively measured rSO_2_ from the start of ALS in consecutive OHCA patients. One sensor (Equanox™ 7600; Nonin) was applied on the patient's forehead's right side when the medical emergency team arrived at the OHCA setting. ROSC was defined as ROSC >20 minutes. Retrospectively, included patients were divided into two groups with respect to their presumed origin of arrest.

## Results

Between December 2011 and October 2014, 113 OHCA patients were included. We observed a significant difference in asystole and VF as initial rhythm between NSc and NSnc, respectively (*P *= 0.035 and *P *= 0.001). In both groups of NS, duration of ALS was significant longer compared with the two S groups (*P *= 0.001 in both comparisons). We observed no significant difference in first measured rSO_2_, mean rSO_2_ until 1 minute before ROSC and increase in rSO_2_ until 1 minute before ROSC (respectively *P *= 0.123, *P *= 0.501, *P *= 0.265) between Sc and Snc. However, when we compare the nonsurvivors of cardiac with noncardiac origin, we observed a significant difference in mean rSO_2 _until 1 minute before ROSC, 35% (27 to 44) in the NSc group and 27 (21 to 34) in the NSnc group (*P *= 0.026). First measured rSO_2_ was 24.5% (13 to 34) in the NSc group and 14 (4 to 28) in the NSnc group (*P *= 0.069) trending to be significantly different. No significant difference was observed in increase until 1 minute before ROSC between both groups of NS (*P *= 0.920). Significant differences was observed in mean rSO_2 _until 1 minute before ROSC and increase in rSO_2_ between Snc and NSnc (*P *= 0.033; *P *< 0.001) and between Sc and NSc (*P *= 0.001; *P *< 0.001).

## Conclusion

We can conclude that NSc have a significant higher mean rSO_2_ and trend to have a significant difference in first measured rSO_2 _compared with NSnc. However, no significant difference was observed between Sc and Snc.

